# (Don’t) Look Up!: Is *short-root* just a short‐root plant?

**DOI:** 10.3389/fpls.2022.1069996

**Published:** 2022-11-17

**Authors:** Eun Kyung Yoon, Jiyeong Oh, Jun Lim

**Affiliations:** Department of Systems Biotechnology, Konkuk University, Seoul, South Korea

**Keywords:** *Arabidopsis*, GRAS transcription factor, shoot development, root development, SCARECROW (SCR), SHORT-ROOT (SHR)

## Abstract

SHORT-ROOT (SHR) is a mobile transcription factor that plays important roles in ground tissue patterning, stem cell niche specification and maintenance, and vascular development in *Arabidopsis* roots. Although mRNA and protein of *SHR* are also found in hypocotyls, inflorescence stems, and leaves, its role in the above-ground organs has been less explored. In most developmental cases, SHR, together with its partner SCARECROW (SCR), regulates the expression of downstream target genes in controlling formative and proliferative cell divisions. Accumulating evidence on the regulatory role of SHR in shoots suggests that SHR may also play key roles in the above-ground organs. Interestingly, recent work has provided new evidence that SHR is also required for cell elongation in the hypocotyl of the etiolated seedling. This suggests that the novel roles of SHR and SHR-mediated regulatory networks can be found in shoots. Furthermore, comparative research on SHR function in roots and shoots will broaden and deepen our understanding of plant growth and development.

## Introduction

Roots of an individual plant play crucial roles in i) acquiring water and nutrients, ii) supporting the plant, iii) synthesizing plant hormones, iv) storing nutrients and metabolites, and v) interacting with soil microbiome (Schiefelbein and Benfey, 1991; Benfey et al., 2010; Petricka et al., 2012). Therefore, understanding the molecular mechanisms controlling root growth and development is of prime importance. Due to the simple cellular organization and a plethora of molecular, genetic, and genomic resources, the model plant *Arabidopsis thaliana* (*Arabidopsis*) has enormously contributed to broadening and deepening our understanding of root growth and development (Schiefelbein and Benfey, 1991; Dolan et al., 1993; Benfey et al., 2010; Petricka et al., 2012).

Three decades ago, in an attempt to isolate mutants with abnormal root structures in *Arabidopsis*, the Philip Benfey lab, then at New York University, identified a mutant that exhibited a short-root growth phenotype (Benfey et al., 1993). Since the recessive mutation resulted in determinate root growth, the mutant was named “*short-root* (*shr*)” (Benfey et al., 1993). In addition to abnormal root growth, detailed phenotypic analyses revealed that *shr* possessed no endodermis, the innermost ground tissue (GT) with the Casparian strip (Benfey et al., 1993). Therefore, *shr* had only a single GT layer between the epidermis and the stele instead of the two layers found in the wild-type (WT) root (Benfey et al., 1993). The root radial pattern defect in *shr* was traced back to the heart-stage embryo (Scheres et al., 1995). Furthermore, when crossed with the *fass* mutant with the multiple GT layers, the endodermis was not restored in the *shr fass* double mutant, indicating that the specification and differentiation of the endodermis depended on SHR function (Scheres et al., 1995).

**Figure 1:**
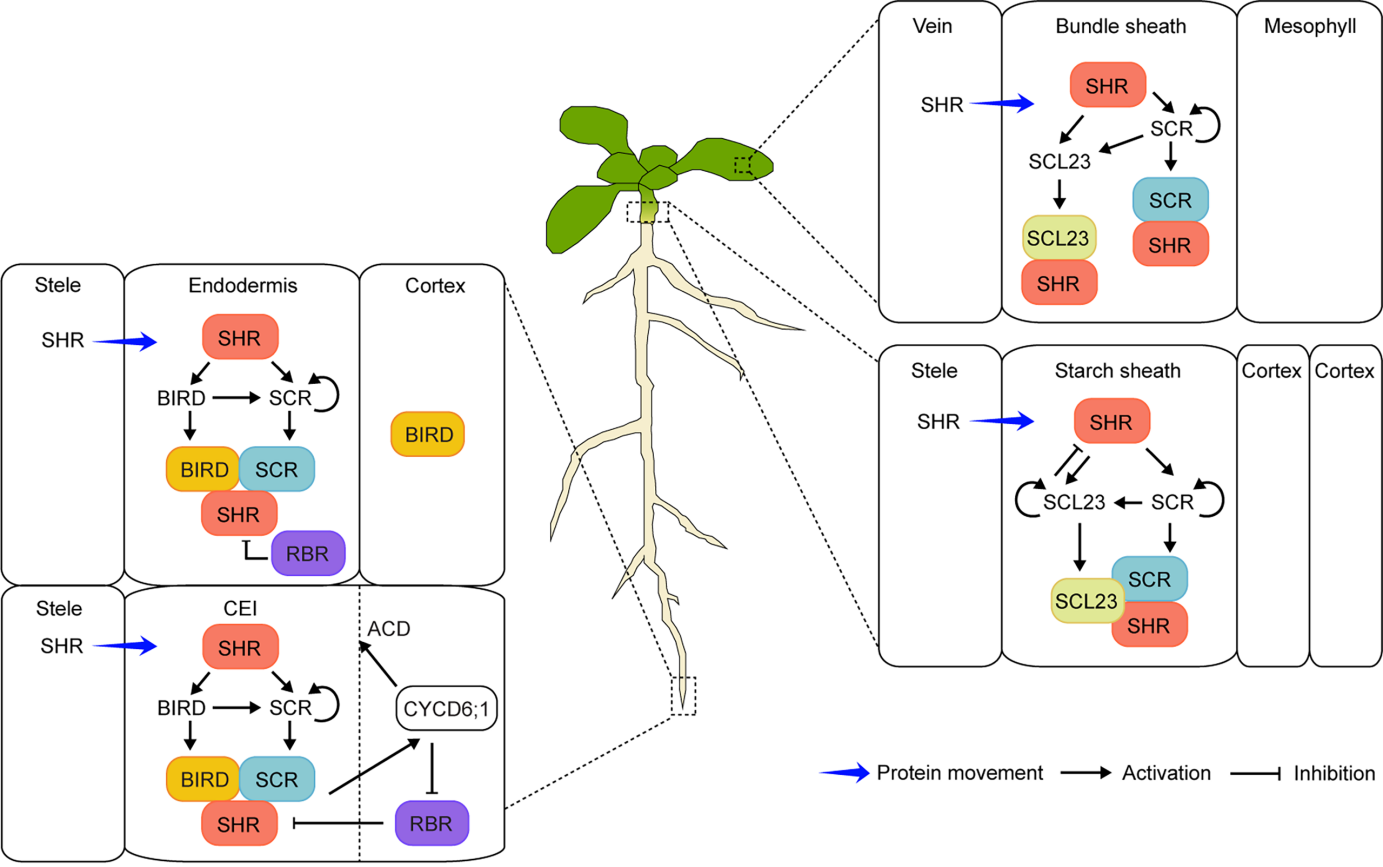
Schematic model of the SHR-mediated regulatory networks in the endodermis development of *Arabidopsis* roots and shoots. In roots (left), SHR protein moves from the stele into the endodermis and CEI (cortex/endodermis initial) where it activates the expression of the downstream target *SCR* and *BIRD* genes. SHR forms protein complexes with SCR and BIRD, resulting in the confinement of SHR in the endodermis and CEI. The protein complexes induce the *CYCD6;1* expression, which subsequently inhibits the negative regulator RBR from interacting with the SHR/SCR complex and promotes the asymmetric cell division (ACD) of CEI. In leaves and hypocotyls (right), SHR as a mobile regulator activates the expression of both *SCR* and *SCL23* in the endodermis and its equivalents (bundle sheath in leaves and starch sheath in hypocotyls). Protein complexes of SHR-SCR, SHR-SCL23, or SHR-SCR-SCL23 can be formed, which prevents SHR from moving beyond the endodermis. Moreover, SCL23 negatively regulates SHR function in hypocotyls.

Similarly, another recessive mutant, *scarecrow* (*scr*), also had one GT layer in roots (Scheres et al., 1995). Unlike *shr*, the remaining GT layer in *scr* showed both endodermis and cortex characteristics, indicating that the periclinal (parallel to the growth axis) formative division to separate the two layers was flawed (Scheres et al., 1995; Di Laurenzio et al., 1996). The *SCR* gene was identified, and its expression was detected in the quiescent center (QC), cortex/endodermis initial (CEI), cortex/endodermis initial daughter (CEID), and endodermis (Di Laurenzio et al., 1996). Four years after *SCR* cloning, the *SHR* gene was also identified and shown to encode a similar transcription factor to SCR, belonging to the GRAS family (Pysh et al, 1999; Helariutta et al., 2000). Interestingly, *SHR* mRNA was observed in the stele. However, the protein moved outward to the tissues (QC, CEI, and CEID) where *SCR* was expressed, indicating that SHR acted as a mobile transcription factor (Helariutta et al., 2000; Nakajima et al., 2001; Gallagher et al., 2004; Gallagher and Benfey, 2009). Moreover, SHR interacted with SCR in the nuclei of the endodermis to control the *SCR* expression for proper radial patterning (Cui et al., 2007; Koizumi et al., 2012a; Koizumi et al., 2012b). In addition to SCR, SCARECROW-LIKE23 (SCL23), the closest SCR homolog, was also shown to play a role in the specification of endodermis cell fate (Long et al., 2015a). Furthermore, JACKDAW (JKD) and its related BIRD transcription factors [also known as INDETERMINATE DOMAIN (IDD)] interacted with SHR to restrict SHR from moving beyond the endodermis (Welch et al., 2007; Long et al., 2015b; Moreno-Risueno et al., 2015; Long et al., 2017; **Figure 1**, left). Other factors, such as RETINOBLASTOMA-RELATED (RBR) and CYCLIN D6;1 (CYCD6;1), also played a role in controlling the formative division to generate the cortex and endodermis (Sozzani et al., 2010; Cruz-Ramírez et al., 2012; **Figure 1**, left). Recently, *SHR* homologs were identified in the roots with multiple GT layers such as date palms, legumes, maize, and *Setaria* (*Setaria viridis*) (Xiao et al., 2019; Dong et al., 2021; Ortiz-Ramírez et al., 2021; Xu et al., 2021; Wang et al., 2022). Indeed, the SHR-mediated regulatory networks also controlled GT formation across species, resulting in generation of a multilayered cortex (Hernández-Coronado and Ortiz-Ramírez, 2021). Unlike *SHR* in the *Arabidopsis* root, all three maize *SHR* homologs (*ZmSHR1*, *ZmSHR2*, and *ZmSHR2-h*) were predominantly expressed in the endodermis, revealed by single-cell RNA sequencing and *in situ* RNA hybridization (Ortiz-Ramírez et al., 2021). Moreover, the ZmSHR1 protein was hypermobile, moving from the endodermis to the cortex layers. Interestingly, the *Zmshr2 Zmshr2-h* double mutant had reduced cortex numbers instead of missing the endodermis, indicating that SHR in maize played a critical role in expansion of the cortex tissue (Ortiz-Ramírez et al., 2021). In addition, its role in cortex multiplication was validated in another monocot *Setaria*, monitored by phenotypic analyses of the loss-of-function mutants of the two *Setaria SHR* homologs (*SvSHR1* and *SvSHR2*). Indeed, the *Svshr1 Svshr2* double mutant showed substantially reduced cortex layers (Ortiz-Ramírez et al., 2021). Therefore, it was suggested that hypermobility of the SHR proteins was common in monocots, which played an important role in multilayered cortex development (Wu et al., 2014; Hernández-Coronado and Ortiz-Ramírez, 2021; Ortiz-Ramírez et al., 2021). Nonetheless, elucidating the role of SHR and its regulatory networks in root radial patterning is still an active subject of research (Zhang et al., 2018; Tian et al., 2022; Yang et al., 2022).

In addition to radial pattern formation, SHR is involved in the specification and maintenance of the root stem cell niche (Sabatini et al., 2003; Qi et al., 2019). The *shr* mutant displayed a loss of functional QC and a reduction of the meristem size, thereby resulting in determinate root growth (Benfey et al., 1993; Helariutta et al., 2000; Sabatini et al., 2003). PLETHORA (PLT) transcription factors were shown to specify and maintain the QC and stem cell niche (Aida et al., 2004; Galinha et al., 2007). However, it was suggested that PLTs and SHR acted in parallel pathways in QC and stem cell niche specification and maintenance (Aida et al., 2004; Galinha et al., 2007; Santuari et al., 2016; Pardal and Heidstra, 2021).

Due to its localization in the root stele (Helariutta et al., 2000; Nakajima et al., 2001), it was reasonable to speculate that SHR might play a role in root vascular development. Indeed, mutations in *SHR* caused reduced cell numbers in the root vasculature (Levesque et al., 2006; Yu et al., 2010) and ectopic metaxylem differentiation in place of protoxylem (Carlsbecker et al., 2010; Yu et al., 2010; Miyashima et al., 2011). For example, SHR and SCR activated the expression of two microRNA (miRNA165 and 166) genes in the endodermis. The resulting miRNA165/166 with gradients restricted their target mRNAs, class III *HOMEODOMAIN LEUCINE ZIPPER* (*HD-ZIP III*) mRNAs at post-transcriptional levels for xylem patterning (Carlsbecker et al., 2010; Miyashima et al., 2011). In addition to xylem patterning, *shr* exhibited severe developmental defects in phloem development (Kim et al., 2020). These studies indicated that SHR non-cell-autonomously exerted its decisive role on the formative cell division for xylem and phloem development. Interestingly, it was demonstrated that SHR controlled cytokinin homeostasis by directly activating the expression of *CYTOKININ OXIDASE3* (*CKX3*) (Cui et al., 2011; Yang et al., 2021). These findings suggested that spatiotemporal regulation of cytokinin levels might be achieved by SHR in the periphery of the root xylem axis (Cui et al., 2011; Yang et al., 2021).

In addition to its role in cell division, recent work revealed that *shr* displayed a drastic reduction in cell elongation in the root maturation zone, suggesting that SHR also played a role in root cell elongation by regulating redox homeostasis (Fu et al., 2021).

Since the first characterization of *shr*, detailed studies have provided insights into its regulatory role in plant roots. Nevertheless, much is still to be learned by unveiling SHR-mediated plant developmental networks.

## Discussion

The very first report of SHR’s involvement in the above-ground organs came from the serendipitous finding that both hypocotyl and inflorescence stem of *shoot gravitropism7* (*sgr7*) displayed no response to a change of gravity vector (Fukaki et al., 1998). The *sgr7* mutant turned out to be allelic to *shr* and had no endodermis/starch sheath in hypocotyls and stems, similar to *shr* roots (Fukaki et al., 1998). In addition, SHR formed protein complexes with SCL23; therefore, the SHR-SCR-SCL23 module played a role in the formation of the functional bundle sheath (also known as endodermis equivalent) in *Arabidopsis* hypocotyls (**Figure 1**, right). These studies indicated that a common molecular mechanism exerted decisive control on the specification and differentiation of the endodermis and its equivalents in shoots and roots (Fukaki et al., 1998; Yoon et al., 2016; Kim et al., 2017).

**Figure 2:**
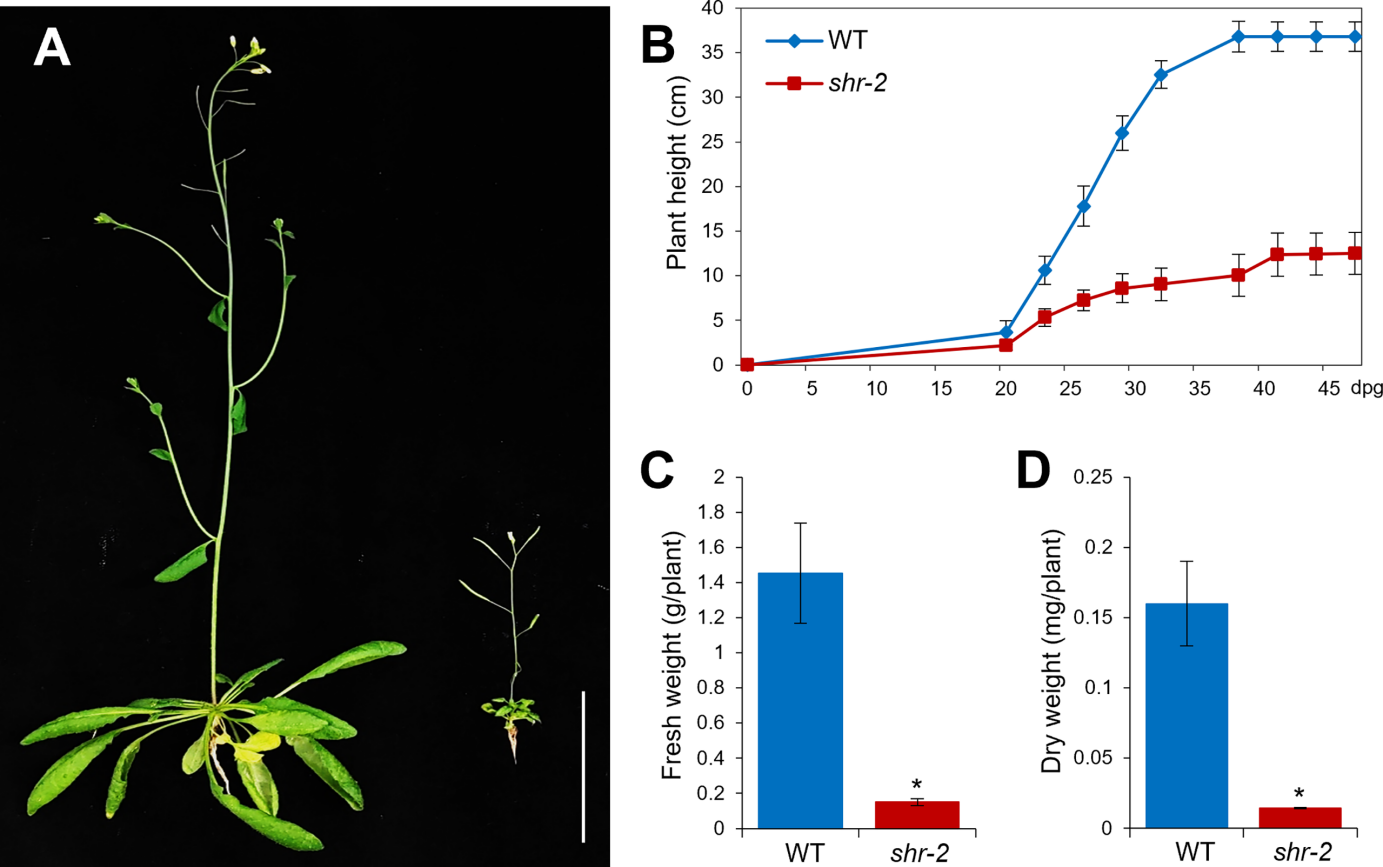
Shoot growth phenotypes of *Arabidopsis* wild-type (WT) and *shr* adult plants. **(A)** Approximately 6-week-old WT and *shr-2* plants. Scale bar: 5 cm. **(B)** Lengths of inflorescence stems of WT and *shr-2* plants at different time points (dpg: days postgermination). **(C)** Fresh weights of ~6-week-old WT and *shr-2* plants. **(D)** Dry weights of ~6-week-old WT and *shr-2* plants. The data are shown as mean ± SEM (n > 30). Statistical significance was determined by Student′s *t*-test compared with WT (*P < 0.05).

Besides the phenotypic perturbations in hypocotyl and stem radial patterning, the shoot growth of *shr* was evidently retarded, thereby resulting in a stunted plant at maturity (**Figures 2A,B**). In addition, *shr* exhibited substantial reductions in fresh and dry weights, which were comparable to approximately one-tenth of the WT levels (**Figures 2C,D**). The identification and characterization of the *SHR* gene and its expression patterns indicated that SHR might play a role in shoot development (Helariutta et al., 2000). Nonetheless, compared to what we have learned about SHR and its regulatory networks in roots, its role in shoots has been less explored.

In leaves, both mRNA and protein of *SHR* were also detected in the vascular bundle and the surrounding bundle sheath (Dhondt et al., 2010; Gardiner et al., 2011; Cui et al., 2014). The size of rosette leaves was severely reduced in *shr* compared to that in WT, suggesting that SHR was involved in proliferative cell division in developing leaves (Dhondt et al., 2010). Moreover, in *shr* leaves, cells in the bundle sheath were rather irregular in shape and became larger than those observed in WT (Cui et al., 2014). Therefore, cells surrounding the vascular core appeared to become mesophyll-like in leaves. As in roots and hypocotyls (Long et al., 2015a; Yoon et al., 2016), the SHR-SCR-SCL23 regulatory module was critically involved in the specification and maintenance of the endodermis equivalent in leaves (Cui et al., 2014; **Figure 1**, right). These studies provided new insights into the role of SHR as a critical regulator in formative and proliferative cell divisions in *Arabidopsis* leaves.

In hypocotyls and stems, *shr* had reduced xylem and phloem areas, resulting in hypocotyls and stems with smaller diameters (Ko et al., 2022). Because the post-transcriptional interaction between miRNA165/166 and *HD-ZIP IIIs* was known to play a crucial role in radial patterning of the shoot vasculature (Emery et al., 2003; Kim et al., 2005), it will be interesting to investigate whether SHR regulates the expression of miRNA165/166 in these organs, as in the root vascular development (Carlsbecker et al., 2010; Miyashima et al., 2011; Kim et al., 2020).

In the etiolated seedling, SHR was also critically involved in controlling hypocotyl cell elongation (Dhar et al., 2022). Hypocotyl cell length in the etiolated *shr* seedling was discernibly reduced, compared to that in WT (Dhar et al., 2022). Indeed, SHR controlled the cell elongation process *via* transcriptional regulation of a group of xyloglucan endotransglucosylase/hydrolase (*XTH*) genes encoding cell wall remodeling enzymes (Dhar et al., 2022). In most cases, SHR acted together with SCR to regulate the expression of downstream target genes (Helariutta et al., 2000; Cui et al., 2007; Carlsbecker et al., 2010; Sozzani et al., 2010; Cruz-Ramírez et al., 2012; Hirano et al., 2017; Long et al., 2017). Unlike the known mode of action, SHR activated the expression of the three *XTH* genes (*XTH18*, *XTH22* and *XTH24*) in a SCR-independent manner (Dhar et al., 2022). SHR is well known to play key roles in regulating formative and proliferative cell divisions. In the etiolated seedling, however, SHR was required for cell elongation. Therefore, this finding indicated that SHR might play previously uncharacterized roles in *Arabidopsis* shoots.

Since the identification and characterization of *shr* were first reported (Benfey et al., 1993), there have been tremendous efforts to isolate homologous genes of *SHR* and elucidate their function in diverse species. Research in monocots, such as rice and maize, provided new insights into SHR’s role in shoots. For example, Kamiya et al. (2003) identified two rice *SHR* homologs (*OsSHR1* and *OsSHR2*) and reported that *OsSHR1* was expressed during stomata development. Likewise, the maize *SHR* homologs (*ZmSHR1* and *ZmSHR2*) were shown to be involved in the development of Kranz anatomy and C_4_ physiology in leaves (Slewinski, 2013; Fouracre et al., 2014; Slewinski et al., 2014; Schuler et al., 2018). In particular, the *Zmshr1* mutant exhibited alterations in patterning and spacing of vascular, bundle sheath and mesophyll cells in maize leaves (Slewinski et al., 2014). Therefore, these reports indicated that the SHR-mediated networks distinctly controlled both vascular and stomata patterning in monocot leaves (Schuler et al., 2018). Thus, it will be interesting to investigate whether SHR also regulates stomata development in *Arabidopsis* leaves.

Although recent studies have identified new regulatory aspects of SHR in shoots, more research is still required (e.g., the interplay between SHR and plant hormones that modulates the growth and development of the above-ground organs at all developmental phases). Together with what we have learned about SHR and its regulatory networks in roots and shoots so far, the time is coming closer to appreciate the whole picture of what role the master regulator SHR plays in plant growth and development. So, it is time to look up!

## Data availability statement

The original contributions presented in the study are included in the article/supplementary material. Further inquiries can be directed to the corresponding author.

## Author contributions

EKY, JO, and JL conceived and designed the research plans. EKY, JO, and JL wrote the manuscript. EKY and JO designed the figures. All authors contributed to the article and approved the submitted version.

## Funding

This work was supported by the Konkuk University Research Fund 2017.

## Acknowledgments

We thank members of the Lim laboratory for comments on the manuscript. We also would like to thank Editage (www.editage.co.kr) for English language editing. We apologize to all colleagues whose work has not been mentioned due to space limitations.

## Conflict of interest

The authors declare that the research was conducted in the absence of any commercial or financial relationships that could be construed as a potential conflict of interest.

## Publisher’s note

All claims expressed in this article are solely those of the authors and do not necessarily represent those of their affiliated organizations, or those of the publisher, the editors and the reviewers. Any product that may be evaluated in this article, or claim that may be made by its manufacturer, is not guaranteed or endorsed by the publisher.
